# Acquired Factor X Deficiency without Amyloidosis Presenting with Massive Hematuria: A Case Report and Review of the Literature

**DOI:** 10.3390/hematolrep15020032

**Published:** 2023-05-15

**Authors:** Sasmith R. Menakuru, Vijaypal S. Dhillon, Ahmed Salih, Amir F. Beirat

**Affiliations:** Department of Internal Medicine, Indiana University School of Medicine, Muncie, IN 47304, USA

**Keywords:** acquired factor X deficiency, COVID-19, hematuria, without amyloidosis

## Abstract

Acquired factor X deficiency is a rare diagnosis, especially without the association of other co-existing conditions such as amyloidosis. The authors report the case of a 34-year-old male with severe frank hematuria found to have markedly prolonged prothrombin time and activated partial thromboplastin time. A mixing study showed correction utilizing normal plasma and a coagulation panel testing revealed decreased factor X activity. The patient was treated with multiple blood transfusions, fresh frozen plasma, high-dose pulse steroids, and rituximab. The patient’s condition improved during his 21-day hospital stay and was followed up every 2 weeks for 3 months. The patient’s factor X level recovered after two weeks of discharge with no other hemorrhagic episodes.

## 1. Introduction

Factor X plays a central role in the coagulation cascade, and is the first enzyme in the common pathway of thrombus formation [1]. Acquired factor X deficiency (AFXD) is a rare condition, with most cases being congenital or due to amyloidosis; however, AFXD without amyloidosis is extremely rare with only a few cases reported in the literature. In 10% of systemic amyloidosis, AFXD is postulated to develop from the absorption of factor X by amyloid fibrils [2]. It is assumed that AFXD is caused by autoimmunity towards FX and, in most cases, such as ours, the specific antibody was unable to be identified. Cases are varied in their presentation and information is limited, but in a portion of reported cases, patients exhibited severe hemorrhagic symptoms with some cases requiring therapeutic intervention. The authors report a case of AFXD in a 34-year-old man with no prior medical history who presented to the hospital with massive hematuria, bruises, and epistaxis requiring multiple blood transfusions. The patient was successfully treated with immunosuppressive therapy with no recurrence since.

## 2. Case Reports

A 34-year-old man was admitted to the hospital for frank hematuria, described as pure blood and recurrent epistaxis, that would not subside. The patient stated his symptoms began while recovering from a severe acute respiratory syndrome coronavirus 2 (SARS-CoV-2/COVID-19) infection that did not require treatment. According to the patient, he did not have any hereditary hemorrhagic conditions or any prior episodes of hemorrhage.

On presentation, physical examination revealed multiple areas of ecchymosis, conjunctival hemorrhage, epistaxis, mucosal bleeding, and massive hematuria. He was febrile to 39.1 °C, tachycardic to 120/min, with normal blood pressure and oxygen saturation. His hemoglobin on admission was 56 g/L (138–172 g/dL) and therefore a unit of blood was administered. Other laboratory tests did not reveal any significant abnormalities, including his platelet count of 240 × 10^9^/L (150–450 10^9^/L).

Coagulation testing revealed both a prolonged prothrombin time (PT) of 190.2 s (11–13.5 s) and an activated partial thromboplastin time (APTT) of 144.9 s (21–35 s). A fibrin degradation product of 3.1 mg/L (<10 mg/L), and D-dimer of 0.22 mg/L (<0.50 mg/L) indicated that the patient was not in disseminated intravascular coagulation (DIC). The fibrinogen level was increased to 5.6 g/L (2.0–4.0 g/L). Computer tomography (CT) of the chest and abdomen/pelvis was unremarkable in terms of any acute findings.

At initial treatment, given his elevated PT and aPTT, it was initially assumed that the clotting disorder was secondary to a developed antibody to a common clotting factor. High-dose methylprednisolone, intravenous immunoglobulin (IVIG) along with a second unit of packed red blood cells (pRBC), and two units of fresh frozen plasma (FFP) were administered. Despite initial treatment, massive hematuria and epistaxis continued.

Given the abnormal coagulation testing, hematology was consulted, who then ordered a mixing study and other pertinent coagulation tests (Table 1). Mixing studies with pooled plasma revealed the correction of the patient’s PT and aPTT, suggesting a factor deficiency. Further supporting a factor deficiency was an increase in the R-value on thromboelastography (TEG). Of the factor tests that were ordered, factor X levels were markedly reduced to 4.7%. To confirm inhibitors were not present, a Bethesda factor X inhibitor titer assay was performed, which was negative. The M-protein, also known as the myeloma protein, which is produced in excess by an abnormal monoclonal proliferation of plasma cells, was not detected in the serum or urine by immunofixation. Furthermore, immunological tests did not show any other abnormalities. The patient was therefore diagnosed as having acquired factor X without amyloidosis.

Multiple case reports were reviewed for treatment options and therefore a consensus was reached to administer a high dose of methylprednisolone at 1 g and rituximab at 375 mg/m² IV. The patient showed improvement with high-dose pulse steroids, rituximab, and IVIG; therefore, plasma therapy was not considered. In total, the patient received 10 units of blood and 35 units of FFP. His clinical symptoms of bleeding slowly improved over the course of his stay with resolution on day 22. The patient was then discharged with a close follow-up with hematology, steroid tapper, and continuation of rituximab for an additional week. The results of PT, aPTT, and FX assays from his biweekly follow-up appointments for three months remained stable and steadily improved to normalcy.

## 3. Discussion

AFXD is an exceedingly rare coagulation disorder, with the subset of AFXD without amyloidosis reported in only about 50 patients in the current literature [3]. Patients present with a sudden onset of bleeding, prolonged measurements of PT and aPTT, and a transient deficiency of factor X. The physiology of amyloidosis causing AFXD is due to the shortened half-life of factor X due to the binding of the factor to amyloid fibrils [4]. The physiology of AFXD without amyloidosis is unknown but assumed to be due to transient inhibitors against factor X [3]. Mulhare et al. demonstrated specific factor X inhibitors may be the cause and postulated that antibodies against microorganisms cross-react with factor X and Xa following an upper respiratory infection; however, these are reported rarely [5]. In our case, a mild infection with COVID-19 infection preceded symptoms.

A review by Lee et al. in 2012 of 34 cases of non-amyloid AFXD showed frequent association with a preceding respiratory infection [6]. Cases that were not preceded by a respiratory infection were associated with lymphoma or multiple myeloma. The initial presentations in these cases ranged from non-bleeding to severe hemorrhagic shock, and a specific inhibitor was observed in only a minority of cases. Many of the cases reviewed presented with gastrointestinal bleeding, with hematuria as the second most common symptom. Nonspecific viral infections preceding the development of AFXD without amyloidosis was seen in 13 of the 34 patients indicating that AFXD may be a post-infectious sequala. The patients were treated with various regimens including corticosteroids, plasma exchange, and IVIG. All of the patients in the cases reviewed by Lee et al. recovered completely, and in some cases, the coagulopathy resolved spontaneously. Ichikawa et al. reviewed recent cases in 2020 that demonstrated similar findings of a previous pulmonary infection [3]. In most cases, there is a component of a previous acute respiratory infection, such as in our case where mild COVID-19 was the pre-existing pathology. In all cases of AFXD without amyloidosis reviewed by Ichikawa et al. AFXD steroids were used. In certain cases, rituximab, chlorambucil, and cyclophosphamide were added to the treatment regimen. The average length of recovery was between three and six weeks. In most reported cases, FX activity was markedly reduced to less than 5%, and patients presented with hemorrhagic symptoms. Most cases were also unable to detect the FX autoantibody, and in those patients where autoantibodies were detected, levels were low. Mixing studies showed correction of both PT and aPTT with normal pooled plasma.

Recognizing acquired AFXD not caused by amyloidosis is difficult, as hemorrhage is nonspecific and can be difficult to diagnose. The authors recommend coagulation studies, a mixing study, and a coagulation panel in patients with hemorrhagic symptoms and prior respiratory infection. AFXD is associated with bleeding in individuals with a factor X activity of less than 10% [7]. The cases are likely to present with bleeding and require supportive care with blood transfusions and fresh frozen plasma. Treatment can consist of high-dose corticosteroids, intravenous immunoglobulins, plasma exchange, and rituximab. Most of the reported cases used plasma exchange, corticosteroids, and intravenous immunoglobulins that revealed adequate responses [3,6]. In serious hemorrhage, the target goal for factor X should be around 40% of normal [8]. Recovery in patients typically occurs in one month but can be prolonged in some cases.

COVID-19 has been associated with thromboembolisms but has rarely been associated with hemorrhagic events, usually explained by disseminated intravascular coagulation and thrombocytopenia. There has only been one other case reported of post-COVID-19-induced AFXD [9]. In the literature, deficiencies of factors V, VIII, XI and XII have been reported due to COVID-19 [10,11,12,13].

In conclusion, the authors hope that clinicians can better recognize the clinical features, diagnosis, and treatment options of acquired AFXD without amyloidosis through this case report. There may be a strong association with a previous respiratory tract infection, and AFXD may be a post-infectious sequela. In patients with a prolonged PT and aPTT with correction by a mixing study, the possibility of an acquired coagulation deficiency should be considered. In patients with coagulation studies revealing a factor X deficiency, other causes of amyloid such as myeloma or oncological first need to be ruled out as they are likely the more common cause. In cases without findings of amyloid-included factor X deficiency, a diagnosis of AFXD without amyloidosis can be made. Due to the rarity of this disease, more research and the study of further cases can lead to a better comprehension of AFXD.

## Figures and Tables

**Table 1:** Pertinent Hematological Values.

Parameter	Laboratory Result	After Mixing Study	Reference Range
Hemoglobin (g/dL)	56		138–172
White Blood Cell (×10^9^/L)	7.9		4.5–11
Platelet Count (×10^9^/L)	240		150–450
PT (s)	190.2	13.1	11–13.5
aPTT (s)	144.9	29.8	21–35 s
Fibrinogen (g/L)	5.6		2.0–4.0
Fibrin Degradation Products	3.1		<10
D-Dimer (mg/L)	0.22		<0.50
Factor II (%)	95.6		80–120
Factor V (%)	99.7		50–150
Factor VII (%)	108		65–140
Factor VIII (%)	101.9		65–130
**Factor X (%)**	**4.7**		**60–140**
Factor XI (%)	78.1		75–145
Factor XII (%)	118.2		50–150
Thromboelastography (TEG)			
R Time (Min)	11.87		5–10
K Time (Min)	3.98		1–5
Maximum Amplitude (mm)	61.7		50–75
Angle (Degree)	56.8		45–75
Lysis at 30 min (%)	0.9		0–10

## Data Availability

Not applicable.
